# Supplementary material for: Integrative genome-wide analyses identify novel loci associated with kidney stones and provide insights into its genetic architecture
Source: Nat Commun. 2023 Nov 18;14:7498. doi: 10.1038/s41467-023-43400-1 (PMC10657403; doi:10.1038/s41467-023-43400-1)
Supplement: Supplementary file 1 — Supplementary information [file 41467_2023_43400_MOESM1_ESM.pdf]

Integrative genome-wide analyses identify novel loci associated with kidney stones and provide insights into its genetic architecture

Supplementary Figures

Supplementary Fig. 1 GWAS meta-analysis of kidney stone disease when unspecified renal colic was included in the cases.

Supplementary Fig. 2 The effect size comparison between our meta-analysis and MGI result.

Supplementary Fig. 3 The expression of *CYP24A1* in different tissues.

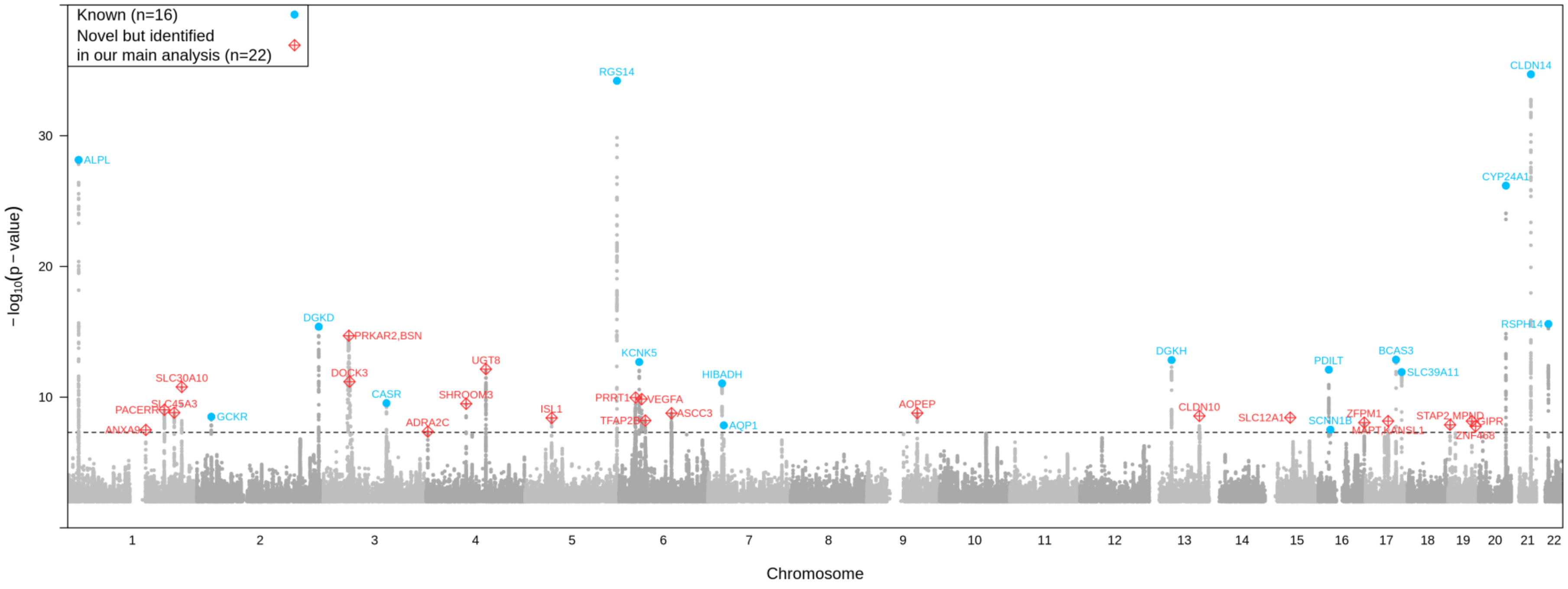

Supplementary Fig. 1 GWAS meta-analysis of kidney stone disease when unspecified renal colic was included in the cases.

Manhattan plot for the *P* values of variants. The grey dash line indicates the genome-wide significance level of 5×10^{-8}. The top variants at the previously identified and novel loci are labeled in blue or red, respectively.

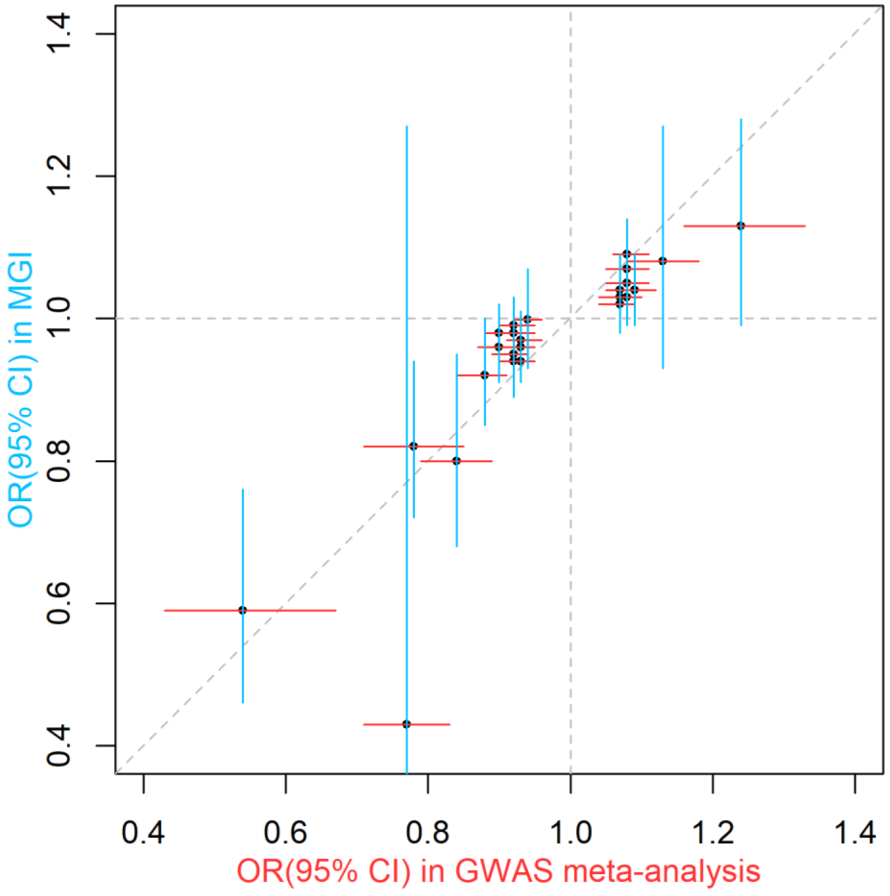

Supplementary Fig. 2 The effect size comparison between our meta-analysis and MGI result.

MGI: Michigan Genomics Initiative project, which includes 6,358 kidney stone disease cases and 43,669 controls.

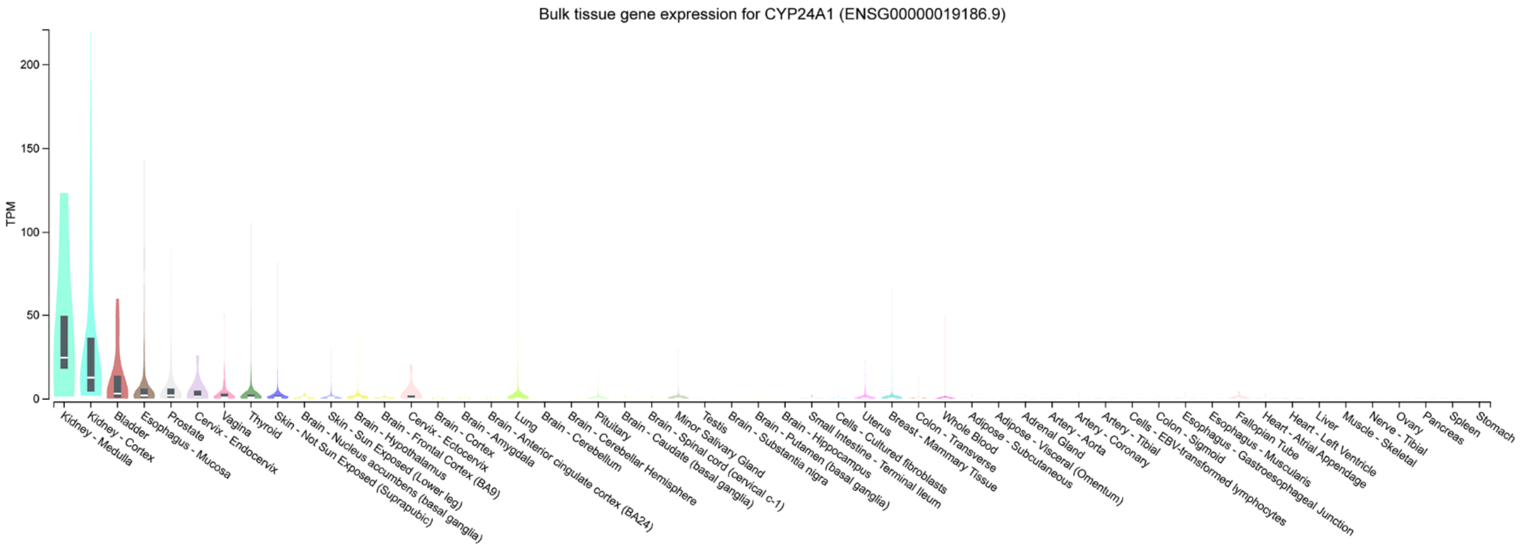

Supplementary Fig. 3 The expression of *CYP24A1* in different tissues.

The y axis represents the expression level with total counts per million (TPM). The x axis represents 54 tissues from the GTEx v.8 project. The color represents different tissue groups.
