# Supplementary material for: Integrative genome-wide analyses identify novel loci associated with kidney stones and provide insights into its genetic architecture
Source: Nat Commun. 2023 Nov 18;14:7498. doi: 10.1038/s41467-023-43400-1 (PMC10657403; doi:10.1038/s41467-023-43400-1)
Supplement: Supplementary file 3 — Description of Additional Supplementary Files [file 41467_2023_43400_MOESM3_ESM.pdf]

Description of Additional Supplementary Files

File Name: Supplementary Data 1
Description: Summary of 56 multiple associated signals at 44 loci with $P < 5 \times 10^{-8}$ in the stepwise conditional analysis

File Name: Supplementary Data 2
Description: The lead variants of GWAS meta-analysis in the known loci

File Name: Supplementary Data 3
Description: Association with blood calcium and phosphate for the independent and significant variants of kidney stone disease

File Name: Supplementary Data 4
Description: Association with 25 hydroxyvitamin D (PMID: 32242144) for the independent and significant variants of kidney stone disease

File Name: Supplementary Data 5
Description: The lead variants of GWAS meta-analysis in the novel loci

File Name: Supplementary Data 6
Description: The significant coding variants

File Name: Supplementary Data 7
Description: Enrichment analysis results of individual cell types by stratified LD score regression

File Name: Supplementary Data 8
Description: The prioritized genes by seven integrative approaches

File Name: Supplementary Data 9
Description: The prioritized genes mapped by kidney-specific eQTL

File Name: Supplementary Data 10
Description: The prioritized genes mapped by kidney-specific meQTL and eQTM

File Name: Supplementary Data 11
Description: The prioritized genes mapped by kidney-specific TWAS

File Name: Supplementary Data 12
Description: The protein expression for the prioritized genes in the kidney from human protein atlas

File Name: Supplementary Data 13
Description: Pathway enrichment analysis.

File Name: Supplementary Data 14
Description: Drug-gene interactions for the prioritized genes

File Name: Supplementary Data 15
Description: Phenome-wide genetic correlations with kidney stone disease
